# Short-term effect of apparent temperature on daily emergency visits for mental and behavioral disorders in Beijing, China: A time-series study

**DOI:** 10.1016/j.scitotenv.2020.139040

**Published:** 2020-09-01

**Authors:** Yanlin Niu, Yuan Gao, Jun Yang, Li Qi, Tao Xue, Moning Guo, Jianpeng Zheng, Feng Lu, Jun Wang, Qiyong Liu

**Affiliations:** a State Key Laboratory of Infectious Disease Prevention and Control, Collaborative Innovation Center for Diagnosis and Treatment of Infectious Diseases, National Institute for Communicable Disease Control and Prevention, Chinese Center for Disease Control and Prevention, Beijing 102206, China; b Beijing Center for Disease Prevention and Control, Institute for Nutrition and Food Hygiene, Beijing 100013, China; c Research Center for Preventive Medicine of Beijing, Beijing 100013, China; d Institute for Environmental and Climate Research, Jinan University, Guangzhou 511443, China; e Guangdong-Hongkong-Macau Joint Laboratory of Collaborative Innovation for Environmental Quality, Guangzhou 511443, China; f Institute of Reproductive and Child Health, Ministry of Health Key Laboratory of Reproductive Health and Department of Epidemiology and Biostatistics, School of Public Health, Peking University, Beijing 100191, China; g Beijing Municipal Health Commission Information Center, Beijing 100034, China; h Beijing Municipal Health Commission Policy Research Center, Beijing 100034, China

**Keywords:** Apparent temperature, Emergency visits, Mental and behavioral disorders, Distributed lag non-linear model

## Abstract

**Background:**

The relationship between temperature and mental disorders is still unclear. This study aims to assess the short-term effect of apparent temperature (AT) on daily emergency visits of mental and behavioral disorders (MDs) in Beijing, China.

**Methods:**

Daily counts of emergency visits related to MDs in Beijing from 2016 to 2018 were obtained. A quasi-Poisson generalized additive model combined with a distributed lag non-linear model (DLNM) was applied to analyze the lag-exposure-response relationship between AT and emergency admissions related to MDs. Sunshine duration, precipitation, PM_2.5_, SO_2_, O_3_, time trend, day of week and holiday were adjusted in the model.

**Results:**

Total daily emergency visits for MDs during the study period were 16,606. With the reference of −2.4 °C (temperature with the minimum emergency visit risk), the single day effects of low AT (−8.6 °C, 10th percentile) and high AT (9.2 °C, 90th percentile) on MDs emergency visits reached a relative risk peak of 1.043 (95%CI: 1.017–1.069) on lag day 4 and 1.105 (95%CI: 1.006–1.215) on lag day 1, respectively. The greatest cumulative effect of high AT emerged on lag 0–5 days and reached a relative risk of 1.435 (95%CI: 1.048–1.965), while no significant cumulative effect of low AT was observed. There was a significant effect of high AT on emergency visits of MDs due to psychoactive substance use and male patients.

**Conclusions:**

Both low and high AT are demonstrated to be the significant risk factors of MDs, which highlights the need of strengthening the health interventions, patient medical services and early warning for patients.

## Highlights

• Both low and high apparent temperature may be the risk factors for emergency visits of psychiatric disorders.
• Much stronger health effect was observed for high apparent temperature.
• Health intervention and medical services are required for patients with psychiatric disorders during days with extreme AT.

## Graphical abstract

**Figure:**
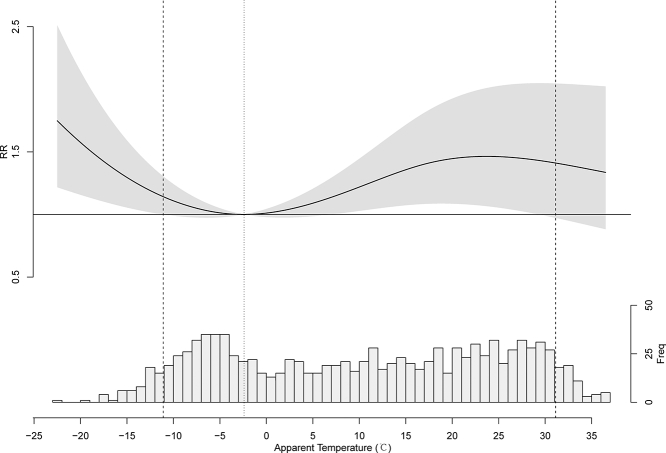

## 1 Introduction

Psychiatric disorders are generally characterized by a combination of abnormal thoughts, perceptions, emotions, behavioral and relationships with others, including neurodevelopmental disorders, bipolar disorders, depressive disorders, anxiety disorders and schizophrenia. A study reported that the global burden of mental illness is underestimated, which accounts for 32.4% of years lived with disability (YLDs) and 13.0% of disability-adjusted life-years (DALYs) (Vigo et al., 2016). In China, the prevalence of psychiatric disorders (excluding dementia) was 16.6% (95% CI: 13.0%–20.2%) during the participants' entire lifetime (Huang et al., 2019) and the adjusted 1-month prevalence was 17.5% (95% CI: 16.6%–18.5%) (Phillips et al., 2009). The total annual costs have increased $3665.4 for individual patients, and $88.8 billion for the whole society in 2013 (Xu et al., 2016).

Previous studies have demonstrated that air pollutants, particularly the particulate matter and nitric oxides, were positively associated with mental health (Buoli et al., 2018; Xue et al., 2019). Vert et al. (2017) found the increasing long-term exposure to air pollution may increase the odds of depression in Barcelona, Spain. These evidences showed the air pollution could potentially mediate the elevated risk of psychiatric disorders, but the further researches focused on a variety of influence factors from the cellular to epidemiological levels are still needed due to the multifactorial nature (Rowland and Majid, 2019; Attademo and Bernardini, 2017; Attademo et al., 2017; Bernardini et al., 2019). In comparison with a range of chronic diseases, such as cerebrovascular, cardiovascular and respiratory disease (Bunker et al., 2016; Yang et al., 2019), increasing attention has been paid on the relationship between temperature and mental health. A study conducted by Peng et al. (2017) in Shanghai, China showed a significant positive association between the temperature above threshold (24.6 °C) and mental disorders hospital admission visits existed at a lag of 0–1 days. Brandl et al. (2018) investigated the influence of weather conditions on the number of psychiatric emergency room patients and found that number of patients in the emergency room increased on warmer and on cloudy days, while lower patient numbers during very cold temperatures. However, Carlsen et al. (2019) found daily number of visits to psychiatric emergency room increased by 22% and 25% when the temperatures were at the 95th percentile and the 5th percentile at lag 0–14 in Sweden, respectively, relative to the seasonal minimum effect temperature. Another study in Lisbon showed a significantly increased risk of hospital admissions of mental and behavioral disorders (MDs) with high temperatures at lag 0–1 and at lag 0–2 days (Almendra et al., 2019). Similarly, Sherbakov et al. (2018) concluded admissions for mental health were significantly increased with higher temperatures in California, US. The study in Italy showed temperature and humidex index were significantly associated with admission in an emergency psychiatric ward, and maximum temperature and solar radiation were associated with bipolar disorder (Aguglia et al., 2020, Aguglia et al., 2019a). Significantly increased risk of the admission counts for schizophrenia was observed at a temperature range of 3.2 °C (10th percentile), and 12.1 °C (99th percentile) in Taiwan, China (Sung et al., 2011). A time-series analysis in Madrid, Spain showed every increase of 1 °C above the threshold temperature (30 °C) could significantly increase the Parkinson's disease related hospital admissions and mortality (Linares et al., 2016). There are also findings suggesting a significant and positive association between the increasing temperature and the risk of suicide (Kim et al., 2019; Gao et al., 2019). However, the existing evidences are mainly for the general psychiatric disorders and suicide, but less for schizophrenia and other disorders.

The mechanism by which the temperature affects the health is extremely complex, as its health impact may be mediated by other weather factors, such as humidity, wind speed and air pressure (Yi et al., 2019a). As a combination of meteorological indicators, including ambient temperature, relative humidity and wind velocity, apparent temperature (AT) has been proven to characterize the physiological experience better than just the temperature alone (Kovats and Hajat, 2008; Steadman, 1984). A study conducted in California, US introduced AT to take into account the combined exposure of temperature and humidity (Basu et al., 2018). AT was also adopted to reflect human thermal to explore the effect of temperature on MDs in Yancheng, China (Min et al., 2019). Though some studies focused on the relationship between temperature and MDs, few researchers used AT as the study variable to quantify its effect on emergency visits of MDs (Peng et al., 2017; Almendra et al., 2019; Yi et al., 2019a; Min et al., 2019; Chan et al., 2018; Wang et al., 2018; Yi et al., 2019b). What's more, most of the study areas in the above studies were located at high latitudes.

However, due to the heterogeneity of contexts in different areas, the study focused on this topic is still limited and the relationship is unclear as well. In this study, we aimed to explore the short-term effect of AT on daily emergency visits of MDs in Beijing, the capital city of China. The evidence may provide implication for the development of health protection strategy, health care services and early warning system of MDs for the policy makers and practitioners.

## 2 Methods

### 2.1 Study area

Beijing is the capital of China with a permanent population of 21.54 million in 2018 (Beijing Statistical Yearbook 2019 published by China Statistics Press). It located in the north of the North China Plain, at latitude 39°56′N and longitude 116°20′E. Beijing has a monsoon-influenced humid continental climate with four distinct seasons, which are characterized by hot, humid summers due to the East Asian monsoon, and cold, windy, dry winters because of the influence of the vast Siberian anticyclone. The annual average temperature is approximately 10–12 °C.

### 2.2 Data collection

Daily data on emergency admissions in 30 hospitals from January 1, 2016 to December 31, 2018 were obtained from Beijing Municipal Health Commission Information Center, in which all registered are covered. Based on the 10th revision of the International Classification of Diseases (ICD-10), records were included if the primary cause of visit was classified related to MDs (F00-F99) and its subtype diseases, including MDs due to Psychoactive Substance Use (F10-F19), Schizophrenia (F20-F29), Mood Disorders (F30-F39) and Neurotic Disorders (F40-F48). In addition to the above diseases, all other MDs were also included as a subtype. Inclusion criteria were: 1) registered permanent residents living in Beijing; 2) the patient with a clear diagnosis based on ICD-10 as listed above. Exclusion criteria were the patient for return visit.

Daily contemporaneous meteorological data were obtained from China Meteorological Data Service Center (http://data.cma.cn/), including the daily mean temperature (°C), daily maximum temperature (°C), daily minimum temperature (°C), relative humidity (%), duration of sunshine (h), barometric pressure (hPa), precipitation (mm), and average wind velocity (m/s). The weather data were measured at three fixed-site stations located in Beijing. Daily mean concentrations of air pollutants at the city level were obtained from China National Environmental Monitoring Centre (http://webinterface.cnemc.cn/), including PM_2.5_ (μg/m^3^), PM_10_ (μg/m^3^), SO_2_ (μg/m^3^), NO_2_ (μg/m^3^), O_3_ (μg/m^3^) (average concentration for 8 h) and CO (μg/m^3^).

### 2.3 Calculation of AT

The AT was calculated by the common meteorological indicators, including daily mean temperature, relative humidity and barometric pressure using the following equations (Krstić, 2011).

(1) AT=T+0.33∗e−0.70∗WS−4.00

(2) $$e=\mathrm{RH}/100*6.105*\exp\left(17.27*T/\left(237.7+T\right)\right)$$

In Eq. (1), T is the daily mean temperature (°C), e water vapor pressure (hPa) and WS average wind velocity (m/s). The water vapor pressure e is calculated with the daily mean temperature and relative humidity using Eq. (2); RH denotes relative humidity (%).

### 2.4 Ethical approval

Ethical approval for the study was obtained from the Chinese Center for Disease Control and Prevention Ethical Review Committee (ICDC-2019008), prior to data collection. All data analyzed were anonymized and under protection by confidentiality agreement. The study was performed in accordance with the Declaration of Helsinki.

### 2.5 Statistical analysis

To assess the distributed lag effect of AT on daily emergency visits of MDs, a quasi-Poisson generalized additive model combined with distributed lag non-linear model (DLNM) was applied, which was motivated by previous studies (Peng et al., 2017; Almendra et al., 2019; Yi et al., 2019a). With the application of the ‘cross-basis’ function (a two-dimensional basis function), DLNM can simultaneously represent the non-linear exposure–response dependencies and delayed effects (Gasparrini, 2014). The model is as follows:

$$Y_{t}\sim \text{quasi}-\text{Poisson}\;\left(\mu_{t}\right)$$

$$\mathrm{Log}\left(\mu_{t}\right)=\alpha +\mathrm{ns}\;\left(\text{time},\mathrm{df}=4*3\right)+{\text{εDOW}}_{t}+{\text{ηHoliday}}_{t}+\mathrm{ns}\left({\mathrm{WF}}_{t},\mathrm{df}=3\right)+\mathrm{ns}\left({\mathrm{AP}}_{t},\mathrm{df}=3\right)+{\mathrm{\beta AT}}_{t,l}$$

where Y_t_ is the daily observed counts of emergency visit for MDs for day t; α is the intercept; ns() is the natural cubic spline. Time as a variable refers to the long-term time trend effect. Moreover, 4 degrees of freedom (df) per year is used for time. Day of the week (DOW) and public holidays were also taken into account as categorical variables, with the corresponding coefficient of ε and η. Weather factors (WF), such as sunshine duration and precipitation, and air pollutants (AP), such as PM_2.5_, SO_2_ and O_3_ were included in the model using a 3 df natural cubic spline, separately. However, considering strong correlation between meteorological indicators and air pollutants (the correlation coefficients over 0.7) (Table S1), we only included the environmental covariates, including duration of sunshine, precipitation, PM_2.5_, SO_2_ and O_3_ in the final model to avoid the bias of multicollinearity. AT_t,l_ is the cross basis matrix of AT; l is the number of lag days; β is the vector of regression coefficients for AT_t,l_. To explore the short-term effect of AT, the maximum lag day of AT is set as 7 days, which was based on the previous studies (Peng et al., 2017; Almendra et al., 2019; Tong et al., 2014). The final composition of the function was a natural cubic spline of AT with 3 df and a natural cubic spline with 3 df for lag days.

In addition, we compared the fitting effect of models adopting AT and daily mean temperature as independent variable, respectively. The quasi Akaike's information criterion (QAIC) was used to measure the relative goodness of fit for the statistical models. A lower QAIC value indicates an improvement in model fit (Gasparrini, 2011). We found AT having the lower value of QAIC based on our data (Table S2). However, there is no significant effect of high temperature on MDs in the model adopting daily mean temperature as independent variable (Fig. S3). Moreover, AT is more easily interpreted to the policy makers and the public. Therefore, AT was adopted in our study.

The AT with the minimum emergency visit risk was −2.4 °C, which was derived from the BLUP (best linear unbiased prediction) of the overall cumulative exposure-response association between AT and MDs emergency visits (Gasparrini et al., 2015) and was taken as a reference of AT in this study. With the reference of AT, the relative risks (RR) with 95% confidence interval (CI) at 10th (defined as low AT) and 90th percentile (defined as high AT) of AT on daily emergency visits of MDs were calculated, respectively.

A stratified analysis by gender and age groups (<18 years, 18–65 years, 66–79 years and ≥80 years) was conducted. Specific diseases were also evaluated in subgroup analysis. All the statistical analyses were performed in R software (3.6.2) with the ‘dlnm’ and ‘splines’ packages. The two-sided P value <0.05 was considered statistically significant.

### 2.6 Sensitivity analysis

To evaluate the robustness of the model, a sensitivity analysis was performed using assessment of several df: time (df = 4–6), AT (df = 3–5), sunshine duration (df = 3–5), precipitation (df = 3–5), PM_2.5_ (df = 3–5), SO_2_ (df = 3–5), O_3_ (df = 3–5). The maximum lag day of AT was also set to 5 and 14 to examine the sensitivity of the cumulative temperature effect.

## 3 Results

During the study period from 2016 to 2018 (1096 days), a total of 16,606 MDs emergency visits were included. Male and patients aged between 18 and 65 years old accounted for 58.98% and 77.29%, respectively. The mean AT and mean temperature were 10.3 °C and 12.1 °C, respectively. The summary of daily MDs emergency visits, meteorological variables and air pollutants are shown in Table 1.

**Table 1:** Descriptive statistics for daily MDs emergency visits, meteorological variables and air pollutants from 2016 to 2018 in Beijing, China.

Group	Sum	Mean ± SD	P10	P25	P50	P75	P90
MDs							
Total	16,606	15.15 ± 5.99	8	11	15	19	23
Male	9794	8.94 ± 3.99	4	6	9	11	14
Female	6812	6.22 ± 3.18	2	4	6	8	10
<18 years	1653	1.51 ± 2.50	0	0	1	2	3
18–65 years	12,835	11.71 ± 5.17	5	8	12	15	18
66–79 years	1198	1.09 ± 1.13	0	0	1	2	3
≥80 years	920	0.84 ± 0.93	0	0	1	1	2
MDs due to psychoactive substance use	2556	2.33 ± 1.82	0	1	2	3	5
Schizophrenia	683	0.62 ± 0.85	0	0	0	1	2
Mood disorders	2076	1.89 ± 1.62	0	1	2	3	4
Neurotic disorders	1950	1.78 ± 2.04	0	0	1	2	4
All other	9341	8.52 ± 4.54	3	5	8	11	14
AT (°C)	–	10.3 ± 14.4	−8.6	−3.8	11.1	23.3	29.2
Tmean (°C)	–	12.1 ± 11.7	−4.1	0.7	13.8	22.9	26.5
Tmax (°C)	–	18.3 ± 11.7	1.9	7.5	19.8	28.8	32.2
Tmin (°C)	–	6.6 ± 11.8	−9.3	−4.5	7.3	17.2	22.0
Duration of sunshine (h)	–	6.93 ± 3.55	1.12	4.4	7.77	9.57	11.1
RH (%)	–	53.42 ± 19.41	28.00	37.33	52.00	70.00	80.00
Precipitation (mm)	–	1.61 ± 7.38	0	0	0	0.03	3.77
BP (hPa)	–	993.75 ± 9.48	981.63	985.54	993.92	1000.99	1006.38
WS (m/s)	–	1.71 ± 0.63	1.03	1.27	1.58	2.00	2.53
PM_2.5_ (μg/m^3^)	–	59.24 ± 54.57	11.78	22.22	43.61	77.56	122.18
PM_10_ (μg/m^3^)	–	89.63 ± 70.46	29.49	44.33	72.28	113.03	167.64
CO (μg/m^3^)	–	0.96 ± 0.76	0.37	0.52	0.79	1.09	1.61
NO_2_ (μg/m^3^)	–	42.74 ± 21.26	21.08	28.04	38.00	52.20	71.54
O_3_ (μg/m^3^)	–	60.55 ± 37.77	14.48	32.21	55.44	83.19	115.66
SO_2_ (μg/m^3^)	–	7.43 ± 7.94	2.05	2.51	4.43	9.03	16.90

P10, the 10th percentile; P25, the 25th percentile; P50, the 50th percentile; P75, the 75th percentile; P90, the 90th percentile; SD, standard deviation; Tmean, daily mean temperature; Tmax, daily maximum temperature; Tmin, daily minimum temperature; RH, relative humidity; BP, barometric pressure; WS, average wind velocity.

**Fig. 1:**
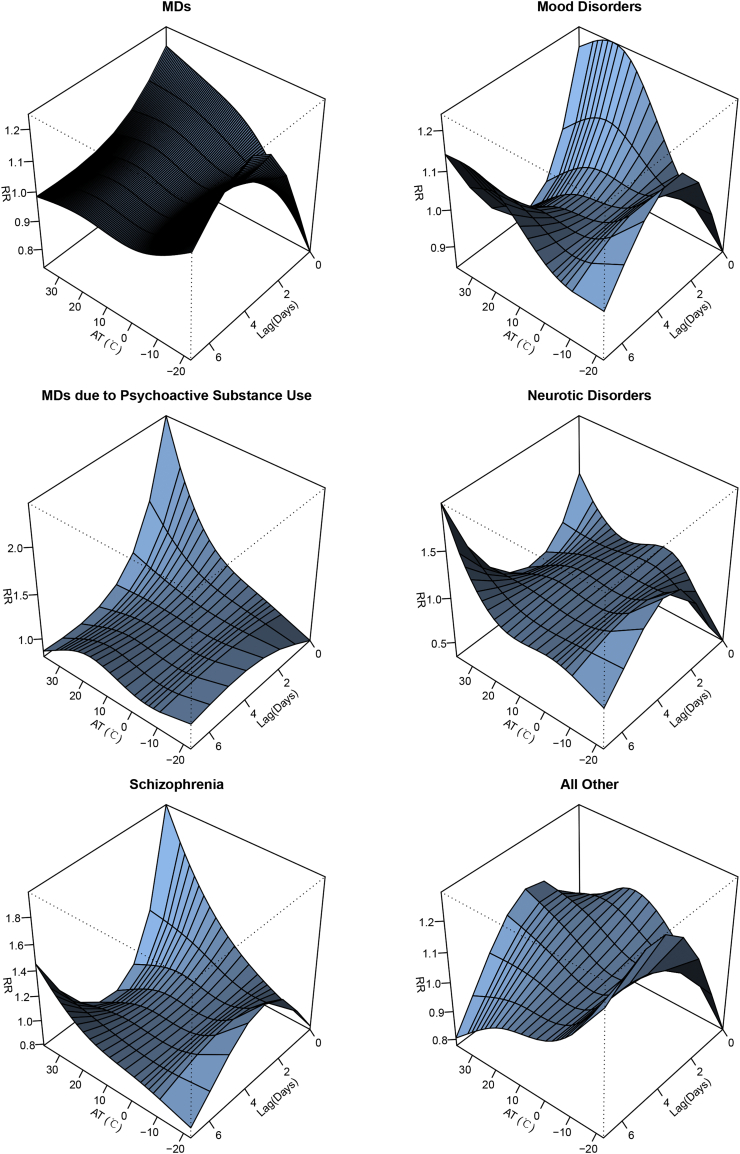
Three-dimension graphs of the relationship between AT and emergency visits for MDs and subtype diseases.

**Fig. 2:**
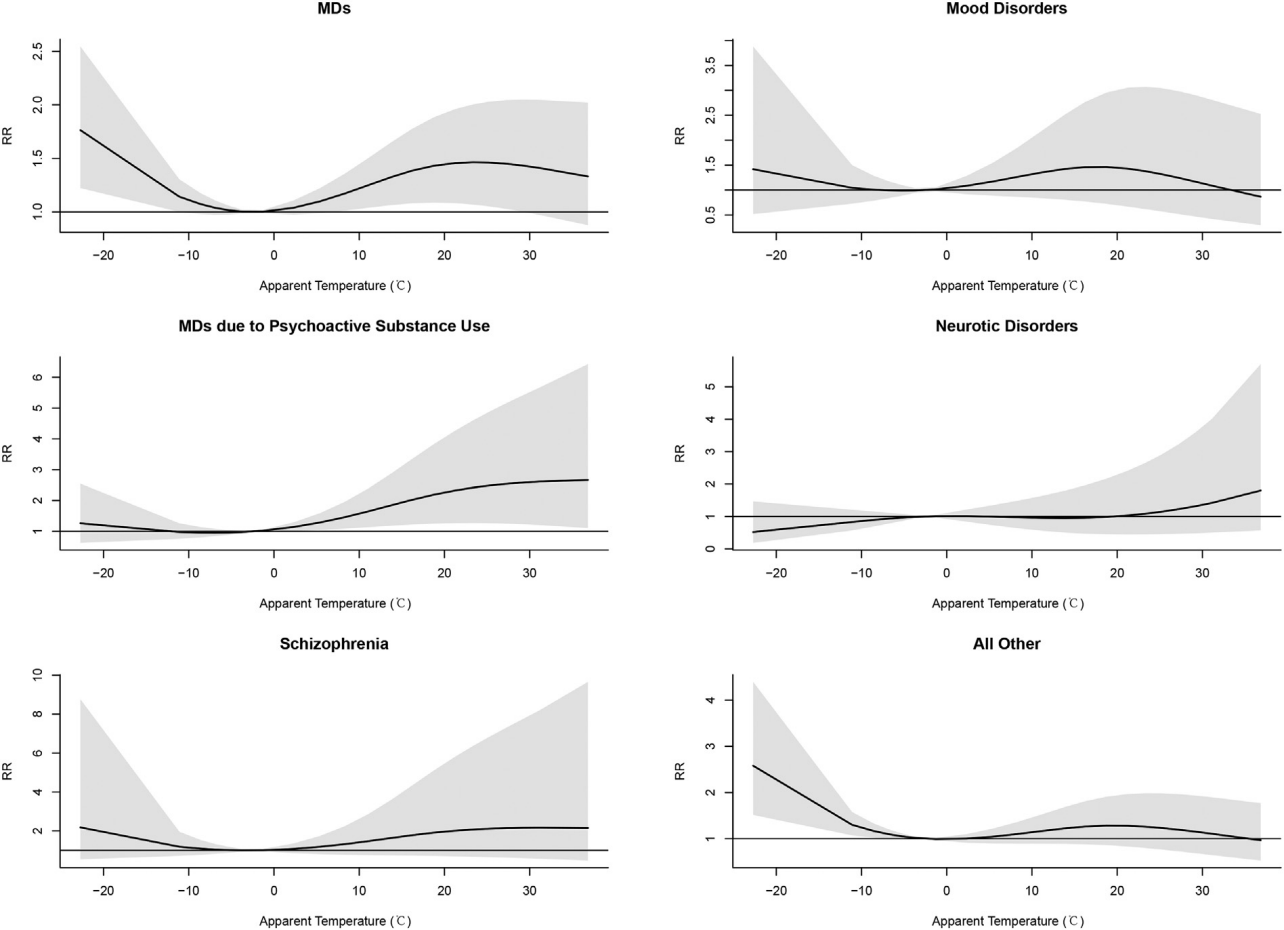
Overall cumulative relative risks (RRs) of AT on emergency visits for MDs and subtype diseases across lag 0–7 days (with 95% CI, shaded gray) in Beijing, China.

Fig. 1 shows the nonlinear relationship between AT and MDs emergency visits along 7 lag days. The overall cumulative RRs of AT on MDs emergency visits cross different lag days manifest that both lower AT and higher AT increased the risk of MDs emergency visits compared to the reference of −2.4 °C (Fig. 2). With the reference of −2.4 °C, the single day and cumulative lag effects of low AT and high AT on MDs emergency visits are showed in Table 2. The effects of low AT on single lag day had statistically significance from the 3rd day to the 6th day, where reached the highest (RR = 1.043, 95%CI: 1.017–1.069) on the lag day 4. However, there was no significant cumulative effect of low AT on MDs emergency visits. For high AT, only on the 1st lag day, there was a single effect (RR = 1.105, 95%CI: 1.006–1.215), while the cumulative effect increased the risk of MDs emergency visits from lag 0–2 days (RR = 1.362, 95%CI: 1.005–1.846) and lasted until lag 0–7 days (RR = 1.433, 95%CI: 1.002–2.049). The greatest cumulative effect emerged on lag 0–5 days, and may increase 43.5% of risk of emergency visits for MDs patients (RR = 1.435, 95%CI: 1.048–1.965). In addition, for high AT, a higher risk was observed than that of low AT, which showed an increased risk of 10.5% for the single effect and 43.5% for the cumulative effect at most.

**Table 2:** The single and cumulative effect estimates of low AT and high AT at different lag day(s) with the reference of −2.4 °C.

Single lag day(s)	RR (95% CI)		Multiple lag day(s)	RR (95% CI)	
	Low AT (−8.6 °C)	High AT (9.2 °C)		Low AT (−8.6 °C)	High AT (9.2 °C)
0	0.925(0.876–0.978)	1.161 (0.939–1.434)	0–0	0.925 (0.876–0.978)	1.161 (0.939–1.434)
1	0.973(0.950–0.997)	1.105 (1.006–1.215)⁎	0–1	0.901 (0.835–0.972)	1.283 (0.954–1.725)
2	1.012(0.989–1.034)	1.062 (0.979–1.151)	0–2	0.911 (0.843–0.984)	1.362 (1.005–1.846)⁎
3	1.034(1.005–1.064)⁎	1.033 (0.931–1.145)	0–3	0.942 (0.873–1.016)	1.407 (1.045–1.895)⁎
4	1.043(1.017–1.069)⁎	1.015 (0.922–1.116)	0–4	0.982 (0.908–1.063)	1.428 (1.052–1.937)⁎
5	1.040(1.023–1.058)⁎	1.005 (0.935–1.080)	0–5	1.022 (0.942–1.109)	1.435 (1.048–1.965)⁎
6	1.031(1.008–1.054)⁎	1.000 (0.916–1.092)	0–6	1.054 (0.971–1.143)	1.435 (1.043–1.975)⁎
7	1.018(0.976–1.062)	0.998 (0.855–1.165)	0–7	1.073 (0.980–1.174)	1.433 (1.002–2.049)⁎

⁎ P < 0.05.

**Fig. 3:**
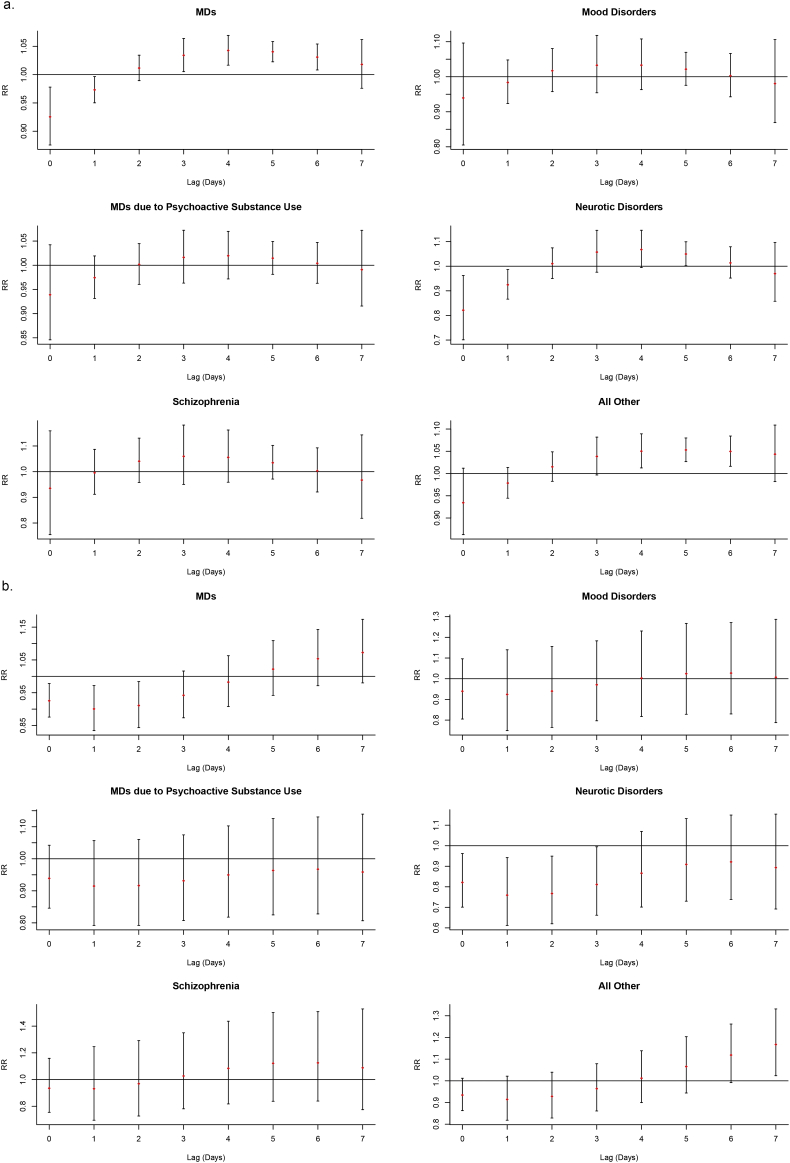
The single (a.) and cumulative (b.) effects of low AT for MDs and subtype diseases cross different lag day(s) with the reference of −2.4 °C.

**Fig. 4:**
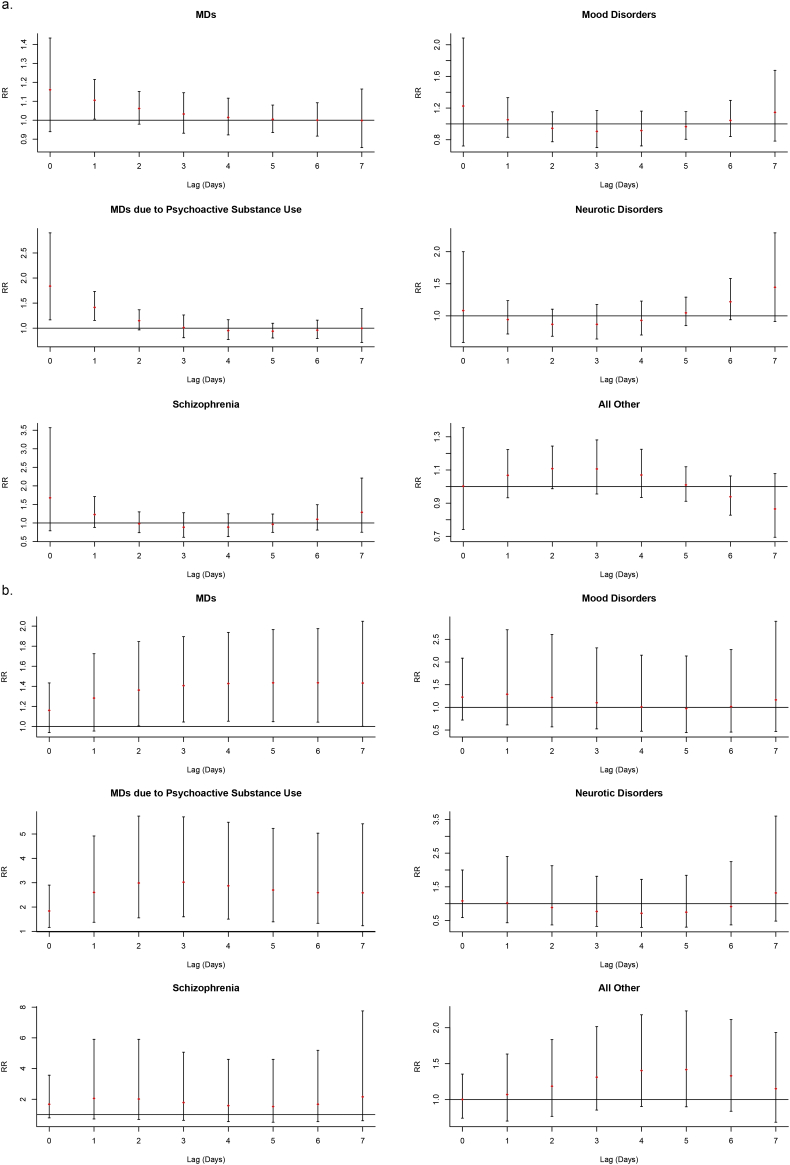
The single (a.) and cumulative (b.) effects of high AT for MDs and subtype diseases cross different lag day(s) with the reference of −2.4 °C.

The result of subgroup analysis showed nonlinear relationships between AT and emergency visits for subtype diseases (Fig. 1). Exposure-response relationships varied in specific diseases, of which MDs due to Psychoactive Substance Use and all other MDs had a significant association with AT (Fig. 2). There was no single day lag effect of low AT on emergency visits for subtype diseases including MDs due to Psychoactive Substance Use, Schizophrenia and Mood Disorders (Fig. 3a). But for Neurotic Disorders and all other MDs, the single day effects were observed on lag 5 day and lag 4–6 days, respectively (Fig. 3a). The highest risk for all other MDs increased 5.3% (RR = 1.053, 95% CI: 1.027–1.080) on the 5th day (Table 3). Regarding the cumulative effect of low AT, only the risk of emergency visits for all other MDs increased by 16.7% on the 7th day after exposure (Fig. 3b and Table S4). While for high AT, the single day effect on the day of exposure and the 1st lag day only had positive correlation with emergency visits of MDs due to Psychoactive Substance Use (Fig. 4a), which increased 83.9% (RR = 1.839, 95% CI: 1.166–2.901) and 41.4% (RR = 1.414, 95% CI: 1.155–1.732), respectively (Table S5). Moreover, for MDs due to Psychoactive Substance Use, the cumulative effects lasted from the day of exposure to the 7th day (Fig. 4b) and the RRs varied from 1.839 (95% CI: 1.166–2.901) on the day of exposure to 3.021 (95% CI: 1.601–5.703) on the 3rd day (Table 4).

**Table 3:** The single day lag effects of low AT at different lag day(s) with the reference of −2.4 °C in different groups.

Low AT	Lag 0	Lag 1	Lag 2	Lag 3	Lag 4	Lag 5	Lag 6	Lag 7
Cause								
MDs	0.925 (0.876–0.978)	0.973 (0.950–0.997)	1.012 (0.989–1.034)	1.034 (1.005–1.064)⁎	1.043 (1.017–1.069)⁎	1.040 (1.023–1.058)⁎	1.031 (1.008–1.054)⁎	1.018 (0.976–1.062)
MDs due to psychoactive substance use	0.939 (0.846–1.042)	0.974 (0.931–1.019)	1.002 (0.961–1.045)	1.016 (0.963–1.072)	1.020 (0.972–1.070)	1.015 (0.981–1.049)	1.004 (0.963–1.047)	0.991 (0.916–1.072)
Schizophrenia	0.935 (0.755–1.159)	0.996 (0.912–1.087)	1.041 (0.958–1.131)	1.060 (0.950–1.181)	1.056 (0.959–1.162)	1.035 (0.971–1.102)	1.003 (0.921–1.093)	0.967 (0.818–1.143)
Mood disorders	0.939 (0.805–1.096)	0.984 (0.923–1.048)	1.017 (0.957–1.081)	1.033 (0.954–1.118)	1.033 (0.963–1.108)	1.022 (0.976–1.070)	1.003 (0.943–1.067)	0.980 (0.869–1.106)
Neurotic disorders	0.821 (0.701–0.962)	0.925 (0.866–0.987)	1.010 (0.950–1.074)	1.057 (0.976–1.146)	1.068 (0.995–1.146)	1.050 (1.002–1.099)⁎	1.013 (0.952–1.079)	0.970 (0.858–1.097)
All other	0.934 (0.863–1.012)	0.979 (0.945–1.014)	1.015 (0.982–1.049)	1.038 (0.997–1.082)	1.050 (1.013–1.089)⁎	1.053 (1.027–1.080)⁎	1.050 (1.016–1.084)⁎	1.043 (0.982–1.109)

Gender								
Male	0.936 (0.877–0.998)	0.980 (0.953–1.008)	1.015 (0.989–1.042)	1.036 (1.002–1.071)⁎	1.043 (1.013–1.074)⁎	1.041 (1.020–1.062)⁎	1.031 (1.005–1.059)⁎	1.019 (0.969–1.071)
Female	0.910 (0.844–0.981)	0.963 (0.932–0.995)	1.006 (0.976–1.037)	1.032 (0.993–1.073)	1.042 (1.007–1.079)⁎	1.040 (1.016–1.065)⁎	1.031 (1.000–1.062)⁎	1.017 (0.960–1.077)

Age groups								
<18 years	0.941 (0.784–1.128)	0.945 (0.867–1.031)	0.957 (0.885–1.034)	0.978 (0.890–1.074)	1.008 (0.927–1.097)	1.046 (0.984–1.113)	1.090 (1.012–1.175)⁎	1.138 (0.996–1.302)
18–65 years	0.932 (0.879–0.989)	0.978 (0.954–1.002)	1.014 (0.990–1.038)	1.035 (1.004–1.067)⁎	1.042 (1.015–1.071)⁎	1.039 (1.021–1.058)⁎	1.029 (1.006–1.054)⁎	1.016 (0.971–1.064)
66–79 years	0.812 (0.684–0.964)	0.954 (0.888–1.025)	1.068 (1.000–1.140)⁎	1.114 (1.022–1.213)⁎	1.096 (1.015–1.182)⁎	1.031 (0.980–1.086)	0.943 (0.880–1.011)	0.850 (0.744–0.970)
≥ 80 years	0.816 (0.682–0.976)	0.943 (0.872–1.019)	1.050 (0.977–1.130)	1.110 (1.012–1.217)⁎	1.123 (1.036–1.217)⁎	1.099 (1.040–1.161)⁎	1.052 (0.977–1.134)	0.996 (0.864–1.149)

⁎ P < 0.05.

**Table 4:** The cumulative effect estimates of high AT at different lag day(s) with the reference of −2.4 °C in different groups.

High AT	Lag 0	Lag 0–1	Lag 0–2	Lag 0–3	Lag 0–4	Lag 0–5	Lag 0–6	Lag 0–7
Cause								
MDs	1.161 (0.939–1.434)	1.283 (0.954–1.725)	1.362 (1.005–1.846)⁎	1.407 (1.045–1.895)⁎	1.428 (1.052–1.937)⁎	1.435 (1.048–1.965)⁎	1.435 (1.043–1.975)⁎	1.433 (1.002–2.049)⁎
MDs due to psychoactive substance use	1.839 (1.166–2.901)⁎	2.601 (1.375–4.919)⁎	2.988 (1.556–5.736)⁎	3.021 (1.601–5.703)⁎	2.873 (1.505–5.484)⁎	2.700 (1.393–5.232)⁎	2.592 (1.335–5.034)⁎	2.585 (1.233–5.419)⁎
Schizophrenia	1.677 (0.788–3.569)	2.059 (0.718–5.906)	2.016 (0.688–5.905)	1.785 (0.629–5.068)	1.585 (0.546–4.600)	1.526 (0.506–4.601)	1.676 (0.541–5.191)	2.158 (0.601–7.755)
Mood disorders	1.226 (0.721–2.085)	1.290 (0.614–2.711)	1.218 (0.570–2.605)	1.103 (0.526–2.312)	1.010 (0.474–2.151)	0.974 (0.445–2.133)	1.017 (0.455–2.274)	1.165 (0.468–2.899)
Neurotic disorders	1.083 (0.586–2.000)	1.020 (0.434–2.401)	0.886 (0.369–2.126)	0.769 (0.326–1.814)	0.715 (0.297–1.722)	0.748 (0.304–1.844)	0.912 (0.370–2.248)	1.319 (0.483–3.600)
All other	1.002 (0.742–1.355)	1.070 (0.701–1.634)	1.186 (0.767–1.834)	1.312 (0.854–2.015)	1.403 (0.904–2.180)	1.418 (0.900–2.234)	1.330 (0.838–2.113)	1.151 (0.685–1.934)

Gender								
Male	1.203 (0.940–1.541)	1.376 (0.973–1.947)	1.505 (1.054–2.148)⁎	1.584 (1.117–2.246)⁎	1.616 (1.131–2.311)⁎	1.606 (1.111–2.322)⁎	1.559 (1.073–2.266)⁎	1.482 (0.974–2.253)
Female	1.102 (0.825–1.471)	1.160 (0.774–1.737)	1.182 (0.781–1.789)	1.188 (0.792–1.783)	1.196 (0.789–1.814)	1.222 (0.796–1.877)	1.275 (0.825–1.970)	1.365 (0.838–2.223)

Age groups								
<18 years	0.994 (0.404–2.447)	1.147 (0.325–4.047)	1.446 (0.396–5.276)	1.818 (0.511–6.461)	2.116 (0.579–7.736)	2.156 (0.572–8.126)	1.853 (0.494–6.954)	1.318 (0.308–5.646)
18–65 years	1.204 (0.970–1.494)	1.334 (0.987–1.805)	1.388 (1.018–1.893)⁎	1.394 (1.029–1.890)⁎	1.383 (1.013–1.890)⁎	1.380 (1.000–1.904)⁎	1.401 (1.009–1.944)⁎	1.454 (1.006–2.102)⁎
66–79 years	1.247 (0.679–2.290)	1.376 (0.587–3.226)	1.406 (0.586–3.376)	1.422 (0.604–3.347)	1.504 (0.628–3.602)	1.733 (0.704–4.267)	2.238 (0.892–5.616)	3.282 (1.160–9.289)⁎
≥80 years	0.839 (0.430–1.637)	0.848 (0.332–2.162)	0.958 (0.366–2.508)	1.086 (0.423–2.787)	1.126 (0.429–2.958)	0.998 (0.369–2.702)	0.723 (0.264–1.983)	0.419 (0.136–1.292)

⁎ P < 0.05.

In gender subgroup, the MDs patients of male and female had the increased RRs of emergency visits at low AT on the lag day 3–6 and the lag day 4–6, respectively, which showed a similar trend for the single day lag effect (Fig. S6a). But in terms of high AT, the single and cumulative effect was only found in male patients and the cumulative effect lasted from lag day 2 to lag day 6 with the highest RR of 1.616 (95% CI: 1.131–2.311) (Fig. S6). There was a positive correlation for single day effect between MDs emergency visits and low AT in all age groups, while the cumulative effect was not significant (Fig. S7). The analysis of single day effects among age groups targeted to high AT showed significant differences in age of 18–65 years and 66–79 years (Fig. S8). While the cumulative effect of high AT on patients aged 18–65 years gradually increased from the 2nd day and reached the greatest risk of 1.454 (95% CI: 1.006–2.102) on the 7th day (Table 4).

The result of sensitivity analysis showed the model was robust when the dfs were altered for the time trend (df = 4–6), AT (df = 3–5), sunshine duration (df = 3–5), precipitation (df = 3–5), PM_2.5_ (df = 3–5), SO_2_ (df = 3–5), O_3_ (df = 3–5) in the mode (Figs. S9–S10). Changing the maximum lag day into 5 and 14 in the model didn't show significant differences for the fitting effect of the model either (Fig. S11). The exposure-response curve was similar before and after adjusting for air pollutants (PM_2.5_, SO_2_ and O_3_) (Fig. S12).

## 4 Discussion

A significantly positive association between AT and MDs emergency visits was found in present study, which means both low and high AT had lag effects on MDs emergency visits with the reference of −2.4 °C and shows a U-shaped curve. Low AT showed a clear single lag day effect on MDs emergency visits between lag day 3 and lag day 6. While the cumulative effect for high AT was much more significant between lag 0–2 days and lag 0–7 days, compared with low AT. Furthermore, both single and cumulative effects of high AT on emergency visits of MDs due to Psychoactive Substance Use were statistically significant. The single day effects of low AT were found in both genders, while high AT had single and cumulative effect only on male patients. Patients with MDs aged 18–65 years and 66–79 years were more sensitive to high AT.

Though the studies focused on the relationship between temperature and MDs are gradually increasing recently, the sound evidence about this is still limited due to the context-specific effect of temperature. To our knowledge, this is the first study aimed to quantify the short-term effect of AT on emergency visits of MDs in Beijing, a typical megacity in northern China. Because of the various methodological settings, we can only compare the results from different studies to some extent. Min et al. (2019) found a positive correlation emerged between high AT and daily emergency admissions of MDs in Yancheng, China, which is consistent with our study. But the single lag effect and the cumulative effect lasted 5 days and 12 days, respectively, which was much longer than that of our study. The possible reasons may be 1) Yancheng is to the south of Beijing with higher AT and mean temperature which may prolong the delayed effect of high AT; 2) the maximum lag day in our study was set to 7, which cannot observe the long-term effect of AT on MDs. Basu et al. (2018) concluded the similar finding that a 5.6 °C increase in same-day AT was associated with 4.8% (95% CI: 3.6%–6.0%) increases in the risk of emergency room visits for MDs. Several studies (Peng et al., 2017; Almendra et al., 2019; Min et al., 2019; Wang et al., 2018; Wang et al., 2014) found the positive correlation between high temperature and MDs but didn't observe the same relationship for low temperature. In our study, the single day effect of low AT emerged from the 3rd day to the 6th day and the maximum increased risk could be 4.3% on the 4th day after exposure. An increased number of psychiatric emergency room at high and low temperatures was also observed by Carlsen et al. (2019) in Sweden. Low temperature was associated with the increase of 18%–25% in psychiatric emergency room but not to a statistically significant extent. In addition, the study in Germany identified high risk of hospital admissions due to MDs at lower temperatures (Shiue et al., 2016). When compared the effects of low and high AT in our study, we found a much stronger effect of high AT on MDs, which has also been demonstrated in most of the related studies that a close correlation between high temperature and MDs existed (Peng et al., 2017; Carlsen et al., 2019; Almendra et al., 2019; Min et al., 2019). We suggested that the difference of effects between low and high temperature needed to be further studied in the future.

Besides all other MDs, the single and cumulative effects of high AT on MDs due to Psychoactive Substance Use were observed in our study. The single day effect emerged on the day of exposure and the 1st day, while the cumulative effect lasted from lag day 0 to lag day 7 with the highest risk of 3.021 (95%CI: 1.601–5.703), which showed significant influence in both magnitude and duration. Psychoactive substance use often leads to elevated heart rate, blood pressure, increased temperature and profuse sweating, which is consistent with serotonin syndrome in symptoms (Gray et al., 2016; Tracy et al., 2017). Simultaneously, heat exposure will aggravate the acute adverse effect caused by psychoactive substance and increase the burden of thermoregulation in body, thereby triggering the onset of the related mental disorders. Some other studies found schizophrenia and mood disorders were strongly associated with temperature (Chan et al., 2018; Yi et al., 2019b), which was not observed in our study. The possible reason for difference among studies may be the limited sample size of subtype diseases in our study. What's more, the sensitivity of specific diseases to temperature may change with settings, which led to the inconsistent conclusions. Related analysis in disease class is scarce due to the data availability, and the biological explanation for the effect of temperature on specific diseases in MDs is also insufficient, which needs to be encouraged in the future.

There was a similar trend that a positive single day effect of low AT on emergency visits of MDs for both genders in our study. While the single and cumulative effects of high AT were significant only in male patients. However, some studies showed different results. For example, a study found the effects of high AT on males and females were similar but the effect of low AT on female was not significant (Min et al., 2019). Wang et al. (2018) found a significant difference in admission rates for schizophrenia between males and females when the temperature increased, with admissions for males greater than those for females. Most studies draw a conclusion that there was effect difference of high temperature on MDs between genders and variations existed among regions but few studied low temperature (Almendra et al., 2019; Wang et al., 2014; Page et al., 2007; Hansen et al., 2008). The physiological function, adaptation ability and living habit may have great difference between genders, which could explain the variations among studies. Studies showed that females were more sensitive to temperature and could become aware of thermal discomfort before males at the beginning of exposure (Lan et al., 2008; Hashiguchi et al., 2010), which could urge them to take preventive measures as soon as possible. From the biological perspective, gonadal hormone plays a great role in gender difference, which may influence the level of neurotransmitters such as serotonin, acetylcholine, norepinephrine involving in the central neuro-molecular thermoregulation (Kelly et al., 1999; David et al., 2001; Lõhmus, 2018). These may be the potential reasons for gender difference in terms of impact of ATs.

An increased risk of low AT on MDs emergency visits was found in all age groups and the effects of high AT in patients aged 18–65 years and 66–79 years were significant. While other studies found the stronger effect of high AT in the patients with MDs aged <45 years (Min et al., 2019), or aged >44 years (Peng et al., 2017), or aged 21–60 years (Wang et al., 2018), or aged >65 years (Liu et al., 2018), which was difficult to be compared with. In addition, Almendra et al. (2019) found no significant differences in effect of temperature between different age groups. The instability of results among studies may due to the non-uniform definition of age groups and the absence of individual socioeconomic factors (Peng et al., 2017). Compared with young adults typical respondence of the old to heat are reduced sweat gland outputs, decreased skin blood flows, reduced cardiac outputs and less redistributions of blood flow from the splanchnic and renal circulations, which increased the heat vulnerability of old individuals (Kenney and Munce, 2003). Furthermore, the elderly usually have underlying diseases, malnutrition and some other adverse health conditions. At this circumstance, the heat exposure can further easily impair the cognitive function of aged population and induce or exacerbate the symptoms of psychiatric disorders. There is also evidence that old individuals held higher susceptibility to cold-induced tau phosphorylation, thereby contributing to an enhanced risk of developing Alzheimer's disease (Tournissac et al., 2017). However, age difference of AT effect needs to be further investigated.

The biological explanations of the relationship between AT and MDs emergency visits may vary for specific disease and are multifactorial. AT is a composite heat index combining ambient temperature, humidity and wind speed. Temperature stress can make direct effect on physio-psychological functions through biochemicals (Chan et al., 2018). Heat stress has a negative association with cognitive function and it may increase plasma serotonin with the inhibition function for the production of dopamine, a neurotransmitter that is responsible for complex task performance (Taylor et al., 2016). Mental illness can increase an individual's physiological vulnerability to temperature by impairing dopaminergic transmission when specific neurotransmitters involved in both of thermoregulation and disease process, which has been demonstrated in animal models (Sung et al., 2011; Bark, 1998; Hasegawa et al., 2005; Davies et al., 2000; Yuan et al., 2006). A study showed that change in the concentration of plasma L-tryptophan, which is a serotonin precursor that negatively associated with depression, had an inverse correlation with suicide (Ogawa et al., 2014; Maes et al., 1995). Kim et al. (2016) reported the concentration of L-tryptophan was negatively associated with ambient temperature. Furthermore, medications in the treatment of MDs may lead to side effects of impaired thermoregulation and suppressed thirst and then increase the risk of emergency visits and hospital admissions at a high temperature (Hasegawa et al., 2005). An uncommon but potential fatal side effect caused by antipsychotic medications is neuroleptic malignant syndrome with the primary reason of dehydration, which is promoted by agitation, poor oral intake, and elevated temperature (Pelonero et al., 1998; Bilanakis et al., 2009). In addition, patients with MDs such as dementia, may lack the awareness to protect themselves from harm of extreme temperature and may not take corresponding actions (Hansen et al., 2008).

AT has been used in other related researches (Basu et al., 2018; Min et al., 2019; Yi et al., 2019b) and was adopted in the model to estimate the effect of temperature on MDs emergency visits in our study. This index performed well in the model fitting with a reasonable explanation to the analysis results. Some researchers also employed physically equivalent temperature (Shiue et al., 2016), temperature variability (Yi et al., 2019a) and temperature range (Sung et al., 2011) to evaluate the relationship between temperature and mental health. Because of the complex effect model of temperature on mental health, an attempt to various temperature-related indicators may be helpful for a deeper understanding of this relationship.

In this study, we controlled the hours of sunlight to avoid its potential influence on relationship between the temperature and psychiatric disorders. Higher sunlight exposure was proved to be positively associated with increasing admissions of bipolar disorder, suicide attempts and other psychiatric disorders (Aguglia et al., 2017, Aguglia et al., 2019b), which could be explained by the damage of the excessive sunlight exposure on biological mechanisms through the dysregulation of serotonin and/or melatonin production or metabolism (Abreu and Bragança, 2015; White et al., 2015).

There were several strengths in the study. First, this is the first study that evaluated the immediate effect and delayed effect of AT on daily MDs emergency visits in Beijing, where is located in relatively high latitudes compared to prior studies in China. This may be one of the reasons why we observed the significant effect of low AT. Second, we analyzed and compared the effects of AT on subtype diseases in MDs in details, which filled the gap to some extent. We concluded that there was a significant association between AT and MDs due to Psychoactive Substance Use.

This study has some limitations. First, both meteorological data and air quality data were station-based monitoring data, which may not reflect the individual exposure precisely and accurately. Second, we were unable to obtain the data regarding social-economic status, disease history and clinical medication of patients with MDs and the contemporaneous traffic noise. This information can be useful for improving the evaluation of the relationship between temperature and MDs. The risk of exposure may also be underestimated because of the lack of the MDs-related self-inflicted injury/suicide data. Third, due to the limited accessible data source, the diagnostic basis of classification adopted in our study is ICD-10, which may lead to inappropriate diagnosis when similar symptoms co-exist. Furthermore, neurotic disorders included in this study include phobic anxiety disorders, panic disorder, generalized anxiety disorder, mixed anxiety and depressive disorder, post-traumatic stress disorder, adjustment disorders, hypochondriacal disorder, neurasthenia and so on, which may show symptoms of minor depression and have some overlaps with depressive disorders. For the similar reason, mood disorder cannot be split into depressive and bipolar disorder, either. This may cause bias for our model to some extent.

## 5 Conclusion

This study demonstrated the significant positive lag effects of both low and high AT on daily emergency visits of MDs. The significant effects of high AT on emergency visits of MDs due to Psychoactive Substance Use and male were observed. Exploration of lag patterns in the effect of AT on MDs showed important public health implications. Based on the understanding of the lag-exposure-response relationship, the policy makers in the health department could provide the early warning for the vulnerable population in time and allocate medical resources reasonably to response to the possible surge in hospital admissions. For individuals, this information prompts them to take preventive measures in advance to decrease the possibility of attack.

## Funding

This work was supported by Strategy and Technology Research on Climate Change Health Risk Assessment, Complex Urban Systems for Sustainability and Health (CUSSH) (Grant Number: 209387/Z/17/Z), the China Prosperity Strategic Programme Fund (SPF) 2015–16 (Project Code: 15LCI1).

## CRediT authorship contribution statement

**Yanlin Niu:** Conceptualization, Formal analysis, Writing - original draft. **Yuan Gao:** Methodology, Software. **Jun Yang:** Methodology, Writing - review & editing. **Li Qi:** Validation. **Tao Xue:** Visualization. **Moning Guo:** Data curation. **Jianpeng Zheng:** Data curation. **Feng Lu:** Investigation. **Jun Wang:** Resources. **Qiyong Liu:** Supervision, Funding acquisition, Project administration.

## Declaration of competing interest

The authors declare that they have no known competing financial interests or personal relationships that could have appeared to influence the work reported in this paper.

## Appendix A Supplementary data

Supplementary material Image 1
