# Supplementary material for: Short-term effect of apparent temperature on daily emergency visits for mental and behavioral disorders in Beijing, China: A time-series study
Source: Sci Total Environ. 2020 Sep 1;733:139040. doi: 10.1016/j.scitotenv.2020.139040 (PMC7298617; doi:10.1016/j.scitotenv.2020.139040)

S1. Spearman’s correlation coefficients of the meteorological indicators and air pollutants

| Variables | AT (℃) | Tmean (℃) | Tmax (℃) | Tmin (℃) | RH (%) | Duration of Sunshine (h) | BP (hPa) | Precipitation (mm) | WS (m/s) | PM2.5 (μg/m^3^) | PM10 (μg/m^3^) | SO2 (μg/m^3^) | NO2 (μg/m^3^) | O3 (μg/m^3^) | CO (μg/m^3^) |
| --- | --- | --- | --- | --- | --- | --- | --- | --- | --- | --- | --- | --- | --- | --- | --- |
| AT (℃) | 1 | 0.99^*^ | 0.97^*^ | 0.99^*^ | 0.55^*^ | 0.08^*^ | -0.87^*^ | 0.41^*^ | -0.24^*^ | 0.10^*^ | 0.04 | -0.51^*^ | -0.24^*^ | 0.68^*^ | -0.06^*^ |
| Tmean (℃) | 0.99^*^ | 1 | 0.98^*^ | 0.98^*^ | 0.47^*^ | 0.14^*^ | -0.88^*^ | 0.36^*^ | -0.18^*^ | 0.06^*^ | 0.03 | -0.50^*^ | -0.26^*^ | 0.72^*^ | -0.11^*^ |
| Tmax (℃) | 0.97^*^ | 0.98^*^ | 1 | 0.93^*^ | 0.41^*^ | 0.25^*^ | -0.88^*^ | 0.30^*^ | -0.18^*^ | 0.06^*^ | 0.06^*^ | -0.45^*^ | -0.21^*^ | 0.72^*^ | -0.12^*^ |
| Tmin (℃) | 0.99^*^ | 0.98^*^ | 0.93^*^ | 1 | 0.56^*^ | -0.01 | -0.85^*^ | 0.46^*^ | -0.21^*^ | 0.08^*^ | 0.01 | -0.53^*^ | -0.29^*^ | 0.67^*^ | -0.08^*^ |
| RH (%) | 0.55^*^ | 0.47^*^ | 0.41^*^ | 0.56^*^ | 1 | -0.50^*^ | -0.39^*^ | 0.55^*^ | -0.65^*^ | 0.41^*^ | 0.17^*^ | -0.28^*^ | 0.16^*^ | 0.01 | 0.44^*^ |
| Duration of Sunshine (h) | 0.08^*^ | 0.14^*^ | 0.25^*^ | -0.01 | -0.50^*^ | 1 | -0.09^*^ | -0.39^*^ | 0.32^*^ | -0.39^*^ | -0.23^*^ | -0.13^*^ | -0.26^*^ | 0.35^*^ | -0.50^*^ |
| BP (hPa) | -0.87^*^ | -0.88^*^ | -0.88^*^ | -0.85^*^ | -0.39^*^ | -0.09^*^ | 1 | -0.36^*^ | 0.12^*^ | -0.16^*^ | -0.15^*^ | 0.36^*^ | 0.17^*^ | -0.66^*^ | 0.03 |
| Precipitation (mm) | 0.41^*^ | 0.36^*^ | 0.30^*^ | 0.46^*^ | 0.55^*^ | -0.39^*^ | -0.36^*^ | 1 | -0.16^*^ | 0.03 | -0.11^*^ | -0.35^*^ | -0.21^*^ | 0.18^*^ | 0.03 |
| WS (m/s) | -0.24^*^ | -0.18^*^ | -0.18^*^ | -0.21^*^ | -0.65^*^ | 0.32^*^ | 0.12^*^ | -0.16^*^ | 1 | -0.40^*^ | -0.24^*^ | 0.01 | -0.47^*^ | 0.24^*^ | -0.49^*^ |
| PM2.5 (μg/m3) | 0.10^*^ | 0.06^*^ | 0.06^*^ | 0.08^*^ | 0.41^*^ | -0.39^*^ | -0.16^*^ | 0.03 | -0.40^*^ | 1 | 0.89^*^ | 0.47^*^ | 0.68^*^ | -0.09^*^ | 0.86^*^ |
| PM10 (μg/m3) | 0.04 | 0.03 | 0.06^*^ | 0.01 | 0.17^*^ | -0.23^*^ | -0.15^*^ | -0.11^*^ | -0.24^*^ | 0.89^*^ | 1 | 0.51^*^ | 0.68^*^ | -0.05 | 0.70^*^ |
| SO2 (μg/m3) | -0.51^*^ | -0.50^*^ | -0.45^*^ | -0.53^*^ | -0.28^*^ | -0.13^*^ | 0.36^*^ | -0.35^*^ | 0.01 | 0.47^*^ | 0.51^*^ | 1 | 0.63^*^ | -0.33^*^ | 0.56^*^ |
| NO2 (μg/m3) | -0.24^*^ | -0.26^*^ | -0.21^*^ | -0.29^*^ | 0.16^*^ | -0.26^*^ | 0.17^*^ | -0.21^*^ | -0.47^*^ | 0.68^*^ | 0.68^*^ | 0.63^*^ | 1 | -0.51^*^ | 0.72^*^ |
| O3 (μg/m3) | 0.68^*^ | 0.72^*^ | 0.72^*^ | 0.67^*^ | 0.01 | 0.35^*^ | -0.66^*^ | 0.18^*^ | 0.24^*^ | -0.09^*^ | -0.05 | -0.33^*^ | -0.51^*^ | 1 | -0.31^*^ |
| CO (μg/m3) | -0.06^*^ | -0.11^*^ | -0.12^*^ | -0.08^*^ | 0.44^*^ | -0.50^*^ | 0.03 | 0.03 | -0.49^*^ | 0.86^*^ | 0.70^*^ | 0.56^*^ | 0.72^*^ | -0.31^*^ | 1 |

* *P＜0.05*. Tmean, daily mean temperature; Tmax, daily maximum temperature; Tmin, daily minimum temperature; RH, relative humidity; BP, barometric pressure; WS, average wind velocity.

S2. Fitting effects of two models were compared using QAIC.

| Model | Deviance residuals | | | | | Residual deviance | QAIC |
| --- | --- | --- | --- | --- | --- | --- | --- |
|  | Min | P25 | Median | P75 | Max |  |  |
| Model 1 | -3.9753 | -0.9148 | -0.1073 | 0.7594 | 5.4343 | 1915.6 | 6932.508 |
| Model 2 | -3.9073 | -0.9151 | -0.1121 | 0.7412 | 5.3448 | 1929.4 | 6947.353 |

Model 1, the DLNM model adopting AT as independent variable; Model 2, the DLNM model adopting daily mean temperature as independent variable; QAIC, Quasi Akaike Information Criterion.

S3. Comparison between models adopting AT and daily mean temperature as independent variable, respectively


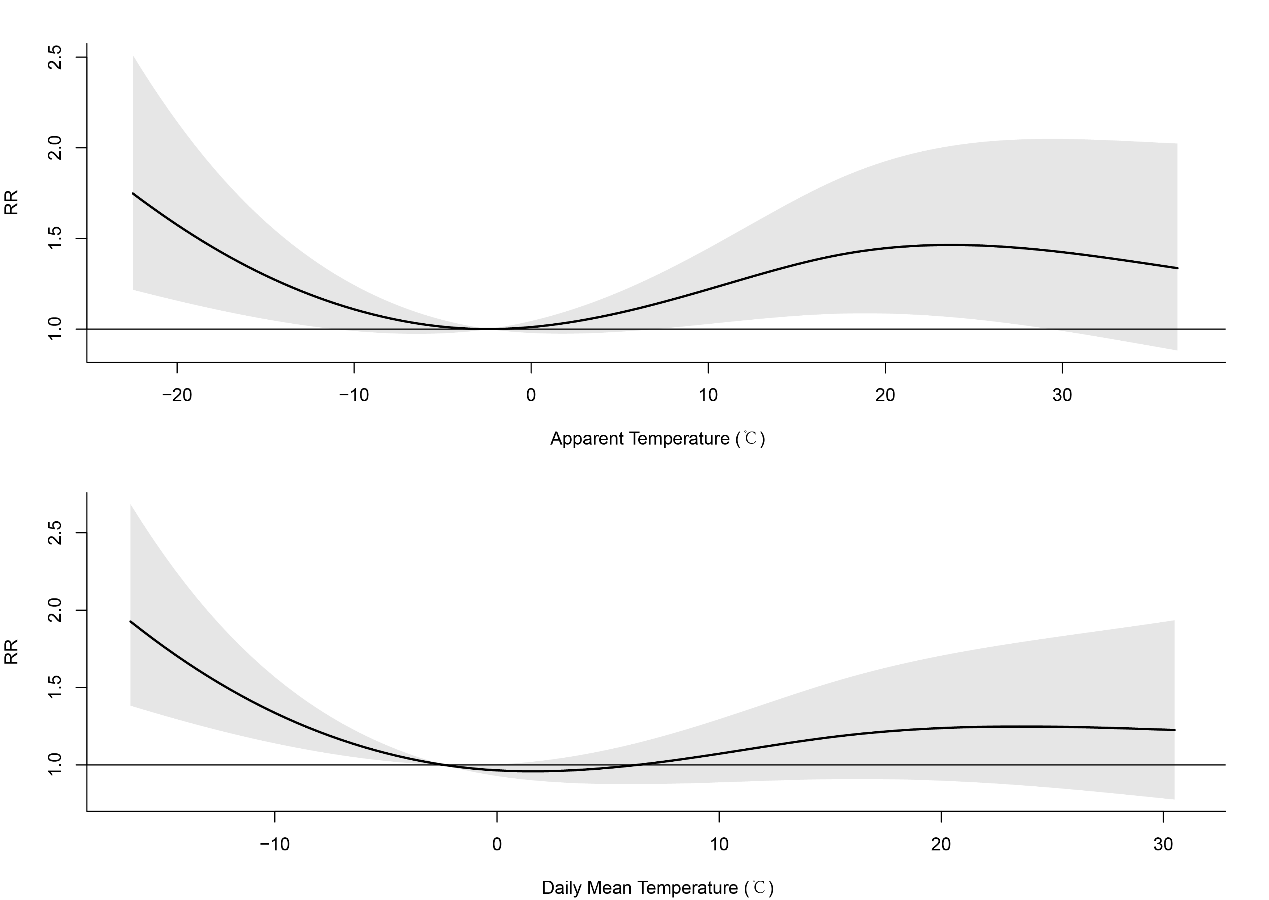


S4. The cumulative effects estimates of low AT at different lag day(s) with the reference of -2.4℃ in different groups

| Low AT | Lag 0 | Lag 0-1 | Lag 0-2 | Lag 0-3 | Lag 0-4 | Lag 0-5 | Lag 0-6 | Lag 0-7 |
| --- | --- | --- | --- | --- | --- | --- | --- | --- |
| Cause |  |  |  |  |  |  |  |  |
| MDs | 0.925(0.876-0.978) | 0.901(0.835-0.972) | 0.911(0.843-0.984) | 0.942(0.873-1.016) | 0.982(0.908-1.063) | 1.022(0.942-1.109) | 1.054(0.971-1.143) | 1.073(0.980-1.174) |
| MDs due to Psychoactive Substance Use | 0.939(0.846-1.042) | 0.915(0.792-1.057) | 0.916(0.792-1.060) | 0.931(0.807-1.075) | 0.950(0.818-1.103) | 0.964(0.825-1.126) | 0.967(0.828-1.131) | 0.959(0.807-1.139) |
| Schizophrenia | 0.935(0.755-1.159) | 0.931(0.696-1.246) | 0.969(0.727-1.291) | 1.027(0.781-1.350) | 1.084(0.818-1.437) | 1.121(0.837-1.502) | 1.125(0.839-1.508) | 1.088(0.775-1.529) |
| Mood Disorders | 0.939(0.805-1.096) | 0.924(0.749-1.140) | 0.940(0.765-1.156) | 0.971(0.797-1.183) | 1.003(0.817-1.231) | 1.024(0.828-1.267) | 1.027(0.830-1.271) | 1.007(0.788-1.287) |
| Neurotic Disorders | 0.821(0.701-0.962) | 0.759(0.612-0.942) | 0.767(0.620-0.949) | 0.811(0.661-0.995) | 0.866(0.701-1.070) | 0.909(0.730-1.132) | 0.921(0.738-1.149) | 0.893(0.692-1.154) |
| All Other | 0.934(0.863-1.012) | 0.914(0.818-1.022) | 0.928(0.828-1.040) | 0.964(0.861-1.079) | 1.012(0.900-1.139) | 1.066(0.944-1.203) | 1.119(0.992-1.262) | 1.167(1.024-1.332) ^*^ |
| Gender |  |  |  |  |  |  |  |  |
| Male | 0.936(0.877-0.998) | 0.917(0.839-1.003) | 0.931(0.851-1.019) | 0.965(0.883-1.054) | 1.006(0.918-1.103) | 1.047(0.952-1.152) | 1.080(0.982-1.188) | 1.100(0.990-1.223) |
| Female | 0.910(0.844-0.981) | 0.876(0.789-0.972) | 0.881(0.793-0.980) | 0.909(0.820-1.009) | 0.948(0.851-1.056) | 0.986(0.882-1.102) | 1.016(0.909-1.136) | 1.033(0.913-1.169) |
| Age Groups |  |  |  |  |  |  |  |  |
| <18 Years | 0.941(0.784-1.128) | 0.889(0.687-1.151) | 0.851(0.647-1.118) | 0.832(0.629-1.101) | 0.839(0.625-1.126) | 0.878(0.649-1.187) | 0.957(0.713-1.285) | 1.090(0.804-1.477) |
| 18-65 Years | 0.932(0.879-0.989) | 0.911(0.841-0.988) | 0.924(0.852-1.002) | 0.956(0.884-1.034) | 0.997(0.918-1.081) | 1.036(0.952-1.127) | 1.066(0.980-1.161) | 1.084(0.985-1.192) |
| 66-79 Years | 0.812(0.684-0.964) | 0.775(0.612-0.981) | 0.827(0.655-1.046) | 0.921(0.736-1.153) | 1.010(0.803-1.270) | 1.041(0.822-1.319) | 0.982(0.774-1.245) | 0.834(0.633-1.099) |
| >=80 Years | 0.816(0.682-0.976) | 0.769(0.600-0.985) | 0.808(0.629-1.038) | 0.897(0.700-1.148) | 1.007(0.778-1.303) | 1.106(0.848-1.444) | 1.164(0.893-1.517) | 1.160(0.861-1.561) |

* *P＜0.05*

S5. The single day lag effects of high AT at different lag day(s) with the reference of -2.4℃ in different groups

| High AT | Lag 0 | Lag 1 | Lag 2 | Lag 3 | Lag 4 | Lag 5 | Lag 6 | Lag 7 |
| --- | --- | --- | --- | --- | --- | --- | --- | --- |
| Cause |  |  |  |  |  |  |  |  |
| MDs | 1.161(0.939-1.434) | 1.105(1.006-1.215) ^*^ | 1.062(0.979-1.151) | 1.033(0.931-1.145) | 1.015(0.922-1.116) | 1.005(0.935-1.080) | 1.000(0.916-1.092) | 0.998(0.855-1.165) |
| MDs due to Psychoactive Substance Use | 1.839(1.166-2.901) ^*^ | 1.414(1.155-1.732) ^*^ | 1.149(0.965-1.367) | 1.011(0.809-1.263) | 0.951(0.774-1.168) | 0.940(0.804-1.098) | 0.960(0.794-1.160) | 0.997(0.715-1.390) |
| Schizophrenia | 1.677(0.788-3.569) | 1.228(0.880-1.712) | 0.979(0.738-1.299) | 0.885(0.614-1.276) | 0.888(0.633-1.246) | 0.963(0.747-1.241) | 1.099(0.809-1.492) | 1.288(0.750-2.210) |
| Mood Disorders | 1.226(0.721-2.085) | 1.052(0.831-1.332) | 0.944(0.774-1.153) | 0.905(0.701-1.169) | 0.916(0.722-1.162) | 0.965(0.806-1.155) | 1.044(0.840-1.298) | 1.146(0.783-1.677) |
| Neurotic Disorders | 1.083(0.586-2.000) | 0.943(0.718-1.238) | 0.868(0.683-1.104) | 0.868(0.639-1.179) | 0.929(0.702-1.230) | 1.047(0.848-1.292) | 1.220(0.940-1.583) | 1.445(0.911-2.293) |
| All Other | 1.002(0.742-1.355) | 1.068(0.932-1.223) | 1.108(0.987-1.244) | 1.106(0.955-1.281) | 1.070(0.934-1.225) | 1.010(0.911-1.119) | 0.939(0.828-1.064) | 0.865(0.694-1.078) |
| Gender |  |  |  |  |  |  |  |  |
| Male | 1.203(0.940-1.541) | 1.144(1.024-1.278) ^*^ | 1.093(0.994-1.202) | 1.053(0.933-1.188) | 1.020(0.913-1.141) | 0.994(0.913-1.081) | 0.971(0.876-1.076) | 0.950(0.793-1.138) |
| Female | 1.102(0.825-1.471) | 1.053(0.925-1.197) | 1.019(0.912-1.138) | 1.005(0.873-1.158) | 1.007(0.884-1.148) | 1.021(0.925-1.127) | 1.044(0.926-1.176) | 1.070(0.867-1.322) |
| Age Groups |  |  |  |  |  |  |  |  |
| <18 Years | 0.994(0.404-2.447) | 1.154(0.772-1.727) | 1.260(0.889-1.787) | 1.257(0.806-1.960) | 1.164(0.773-1.752) | 1.019(0.751-1.383) | 0.859(0.594-1.245) | 0.712(0.370-1.369) |
| 18-65 Years | 1.204(0.970-1.494) | 1.108(1.007-1.221) ^*^ | 1.040(0.958-1.130) | 1.004(0.904-1.116) | 0.992(0.900-1.094) | 0.998(0.927-1.074) | 1.015(0.928-1.11) | 1.038(0.887-1.215) |
| 66-79 Years | 1.247(0.679-2.290) | 1.103(0.841-1.448) | 1.022(0.813-1.285) | 1.011(0.755-1.355) | 1.058(0.806-1.387) | 1.153(0.937-1.418) | 1.291(1.002-1.663) ^*^ | 1.466(0.944-2.279) |
| >=80 Years | 0.839(0.430-1.637) | 1.011(0.750-1.363) | 1.131(0.875-1.460) | 1.133(0.818-1.569) | 1.037(0.768-1.400) | 0.887(0.709-1.109) | 0.724(0.552-0.952) | 0.579(0.357-0.938) |

* *P＜0.05*


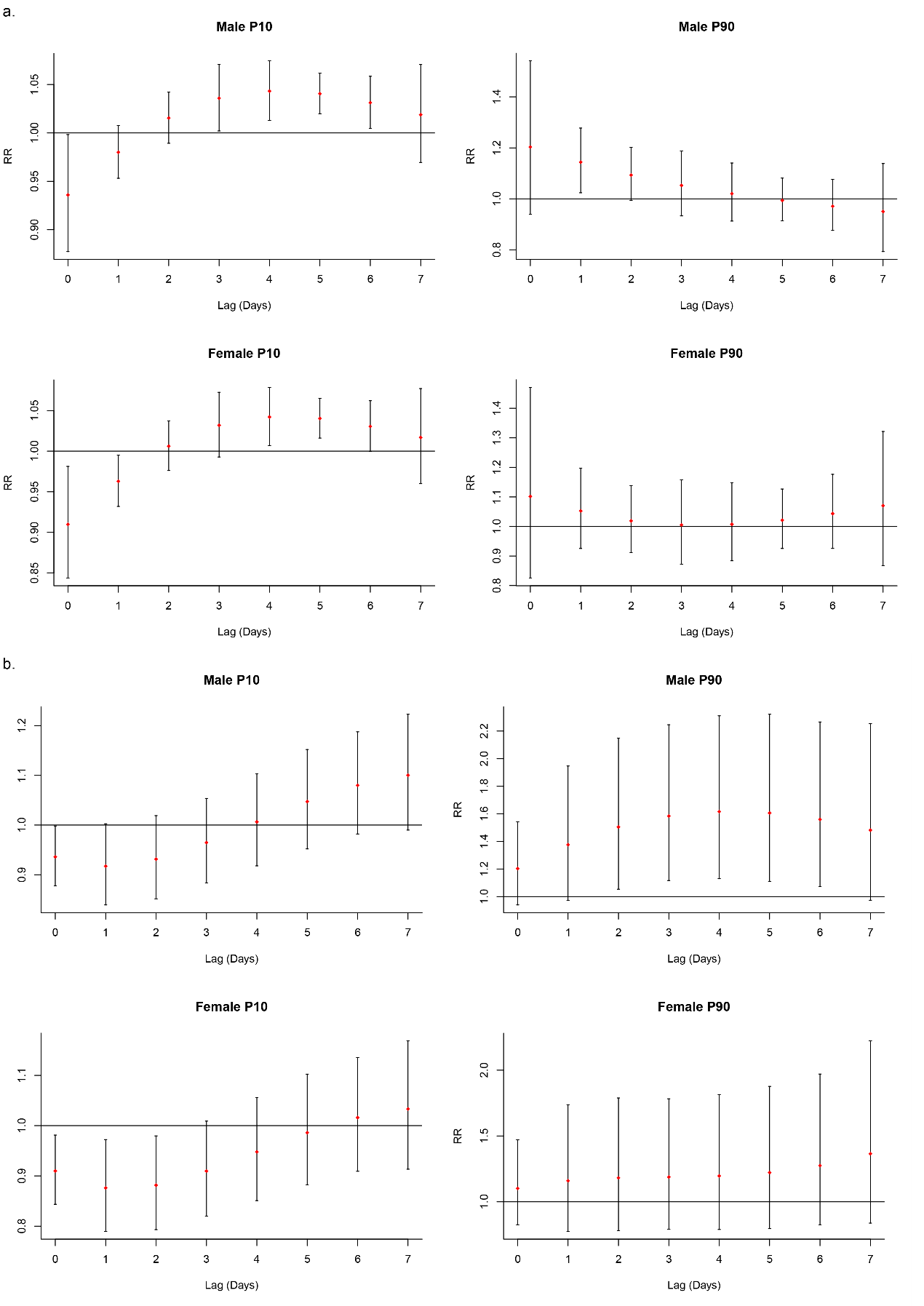


S6. The single (a.) and cumulative (b.) effects of AT for MDs cross different lag day(s) with the reference of -2.4℃ in genders. P10, 10^th^ percentile; P90, 90^th^ percentile.


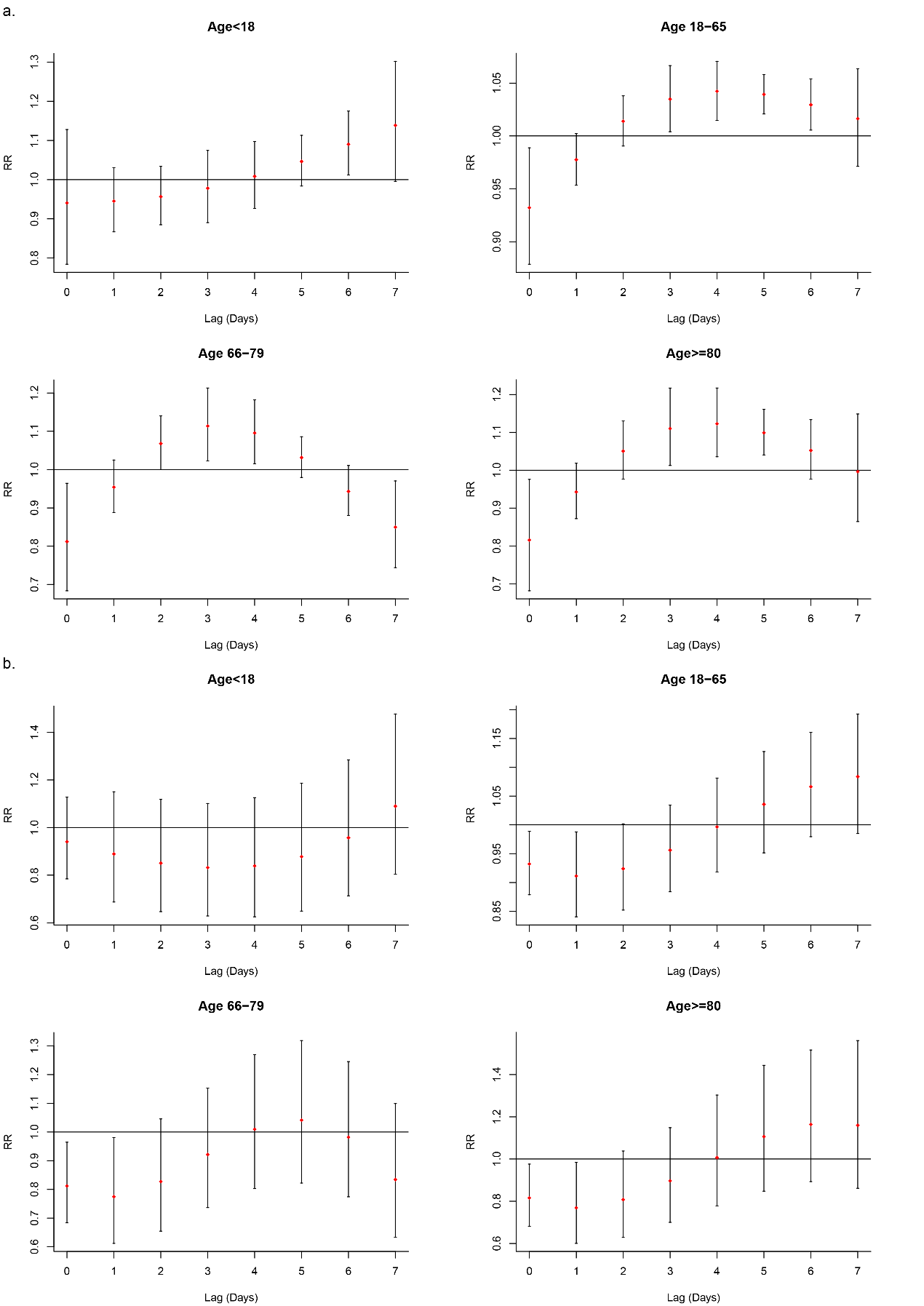


S7. The single (a.) and cumulative (b.) effects of low AT for MDs cross different lag day(s) with the reference of -2.4℃ in age groups


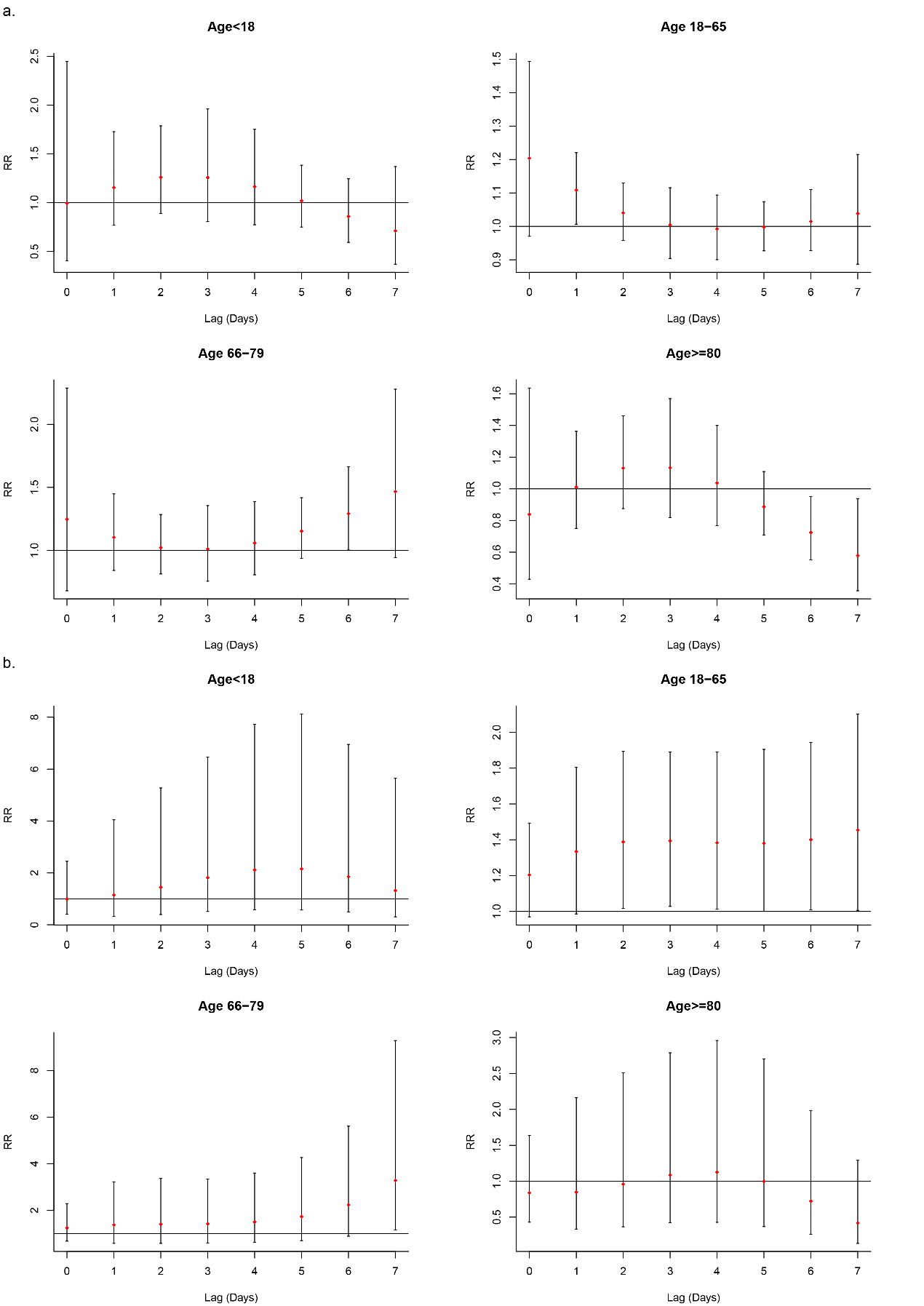


S8. The single (a.) and cumulative (b.) effects of high AT for MDs cross different lag day(s) with the reference of -2.4℃ in age groups

S9. Sensitivity analysis when altering the degrees of freedom (df = 4–6) for controlling for the long-term trend and seasonality in the model


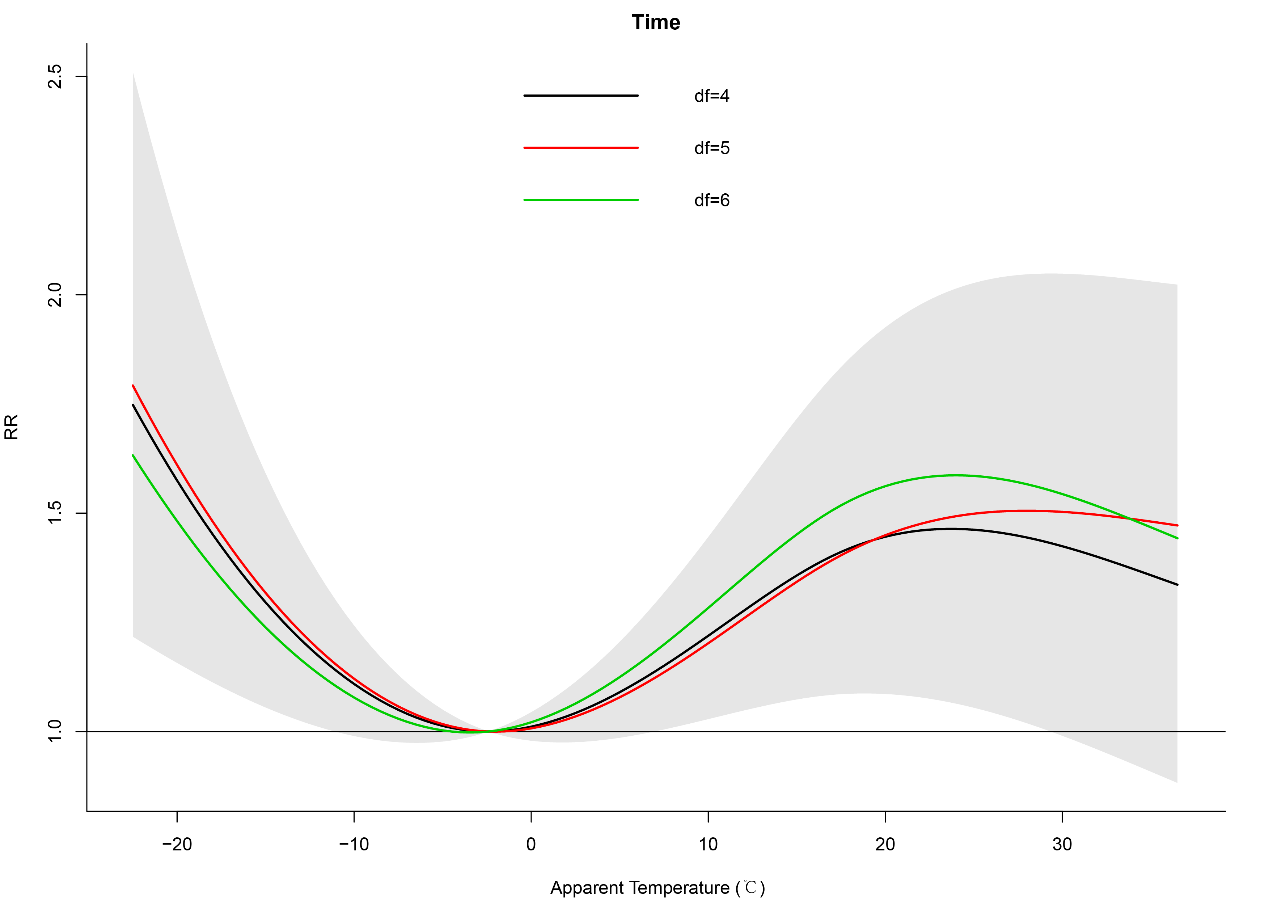


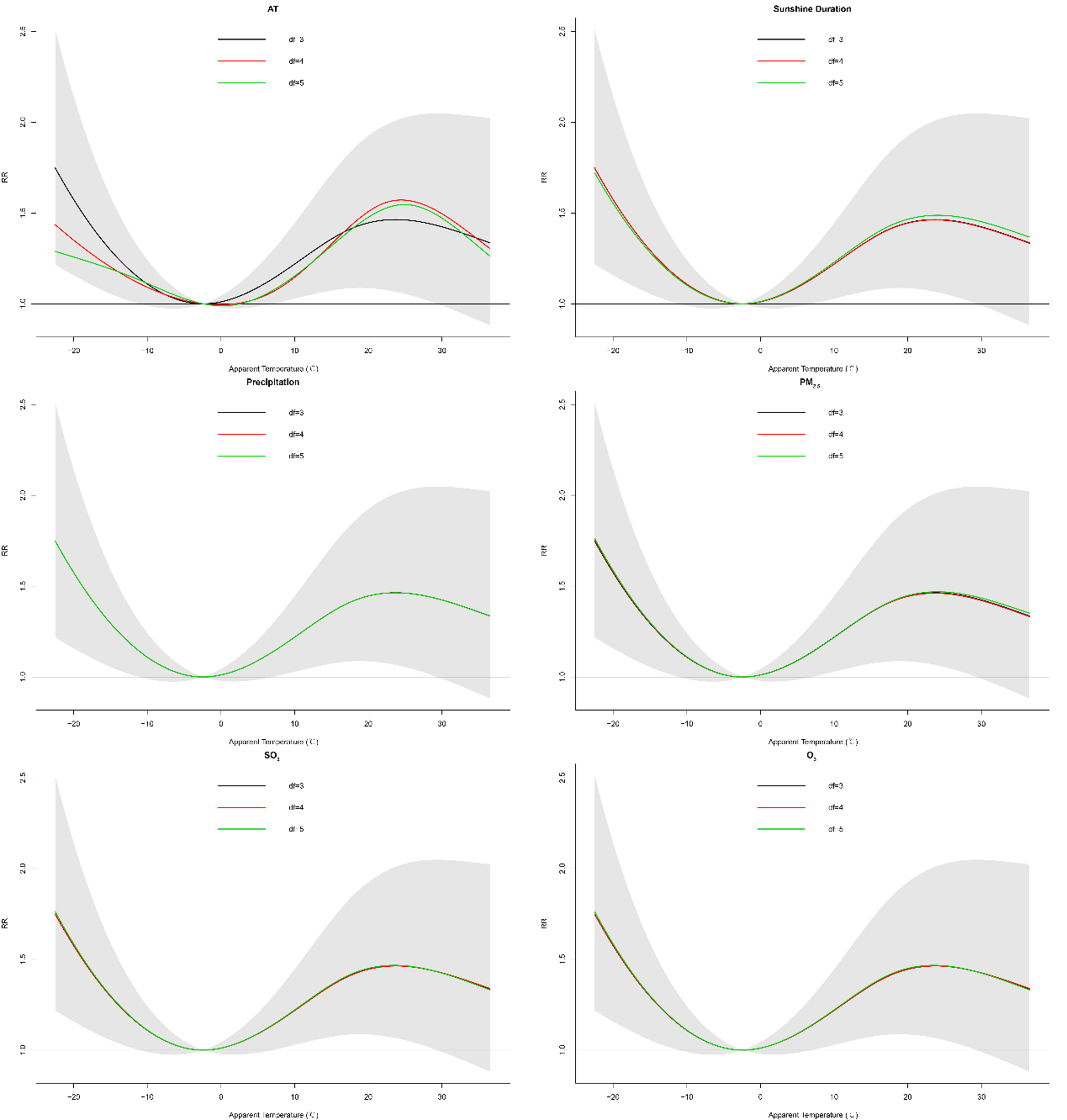
S10. Sensitivity analysis when altering the degrees of freedom (df = 3–5) for AT, sunshine duration, precipitation, PM_2.5_, SO_2_ and O_3_ in the model

S11. Sensitivity analysis when changing the maximum lag day into 5 and 14 in the model


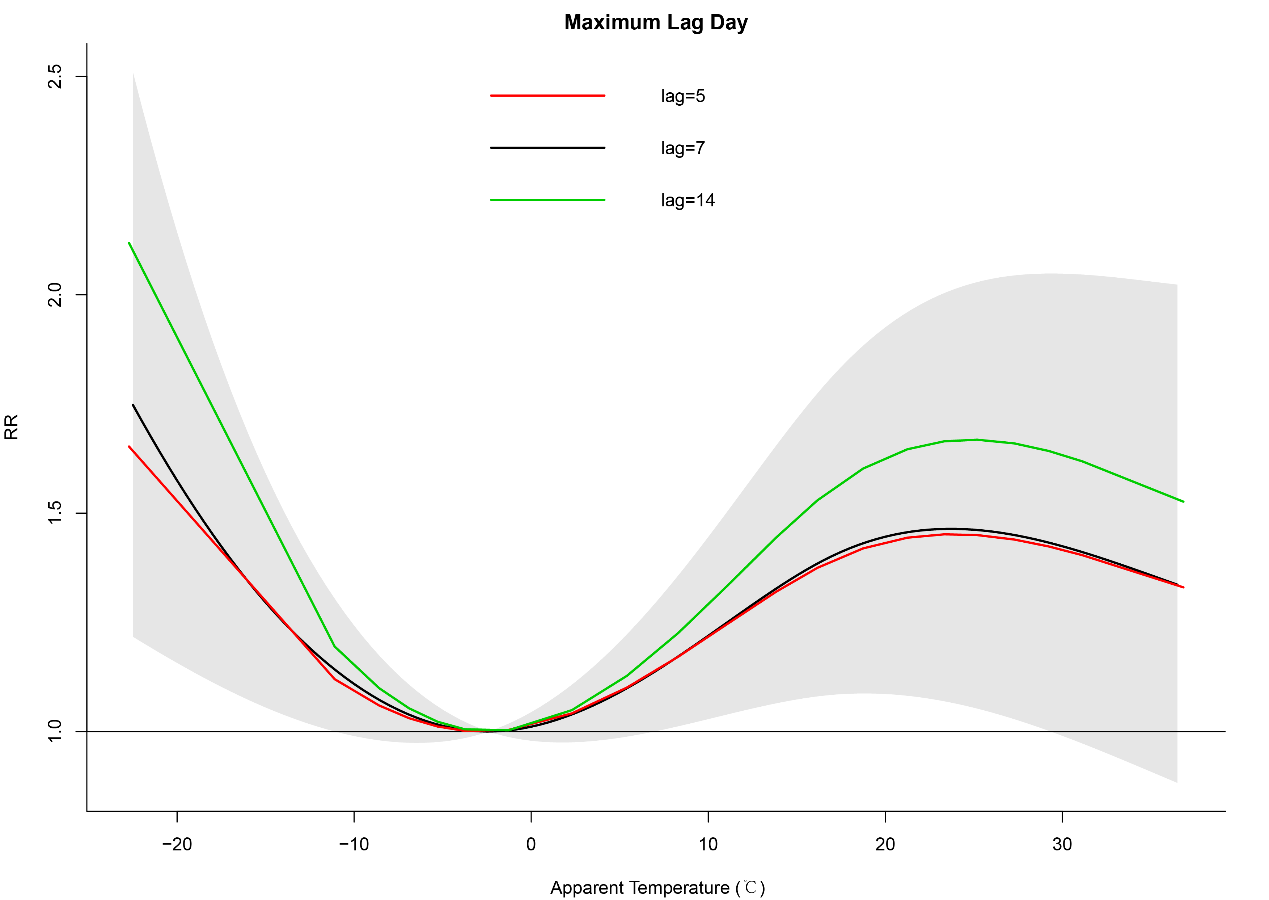


S12. Sensitivity analysis between models with air pollutants and without air pollutants


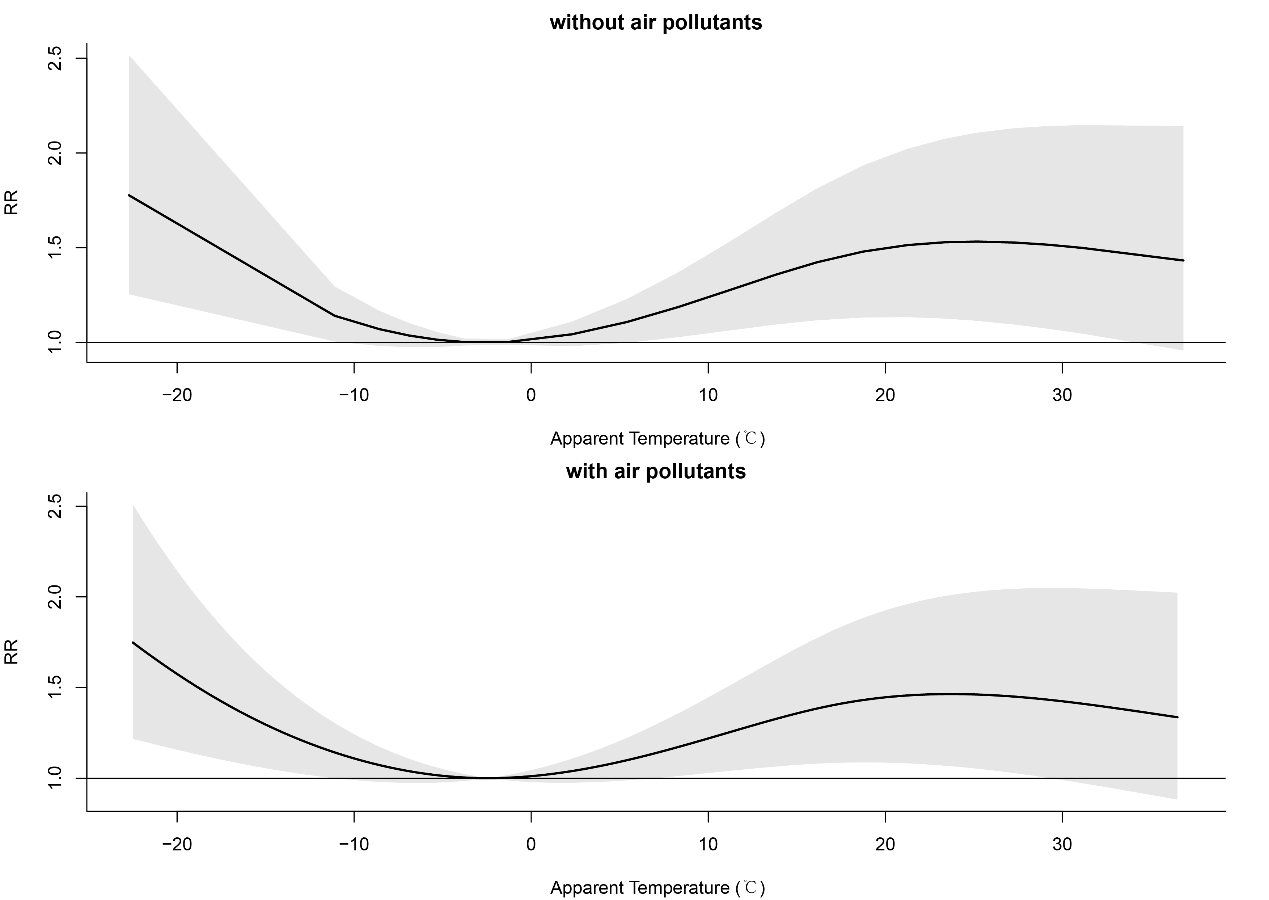

Supplement: Supplementary file 1 — Supplementary material [file mmc1.docx]
